# Clear Cell Carcinoma Arising in an Abdominal Wall Cesarean Section Scar: A Case Report With Description of Pathological and Molecular Features

**DOI:** 10.3389/fsurg.2021.735381

**Published:** 2021-09-14

**Authors:** Cristina Colarossi, Maria Carolina Picardo, Lorenzo Colarossi, Enrica Deiana, Costanza D'Agata, Corrado Fichera, Eleonora Aiello, Giorgio Giannone, Lorenzo Memeo

**Affiliations:** ^1^Pathology Unit, Department of Experimental Oncology, Mediterranean Institute of Oncology, Catania, Italy; ^2^Surgical Oncology Unit, Department of Experimental Oncology, Mediterranean Institute of Oncology, Catania, Italy

**Keywords:** clear cell carcinoma, c-section, PIK3CA, endometriosis, abdominal wall

## Abstract

Clear cell carcinoma is a clinically and biologically distinct type of carcinoma predominantly encountered in the ovary and endometrium. In the ovary, it is frequently associated with endometriosis, which is a well-known risk factor. Endometriosis has often been described in the abdominal wall of women who had a cesarean section; however, malignant transformation is a very rare event, occurring in <1% of cases. The authors report a case involving a 52-year-old woman with an abdominal wall nodule at a cesarean section scar. Radiology revealed a mass, measuring 8 cm in size, in the abdominal wall suggestive of a soft tissue tumor. After resection, histology revealed the presence of clear, eosinophilic, and hobnail cells, which, together with immunohistochemical and molecular findings, enabled the diagnosis of clear cell carcinoma of the abdominal wall. The present report describes the clinical, radiological, pathological, and molecular features of an unusual abdominal lesion that represents a rare but challenging diagnosis.

## Introduction

Clear cell carcinoma (CCC) of the female genital tract is usually localized to the ovary or endometrium. Its localization within an abdominal wall scar is extremely rare, and its pathogenesis is usually related to malignant transformation of endometriosis, which develops after hysterectomy or cesarean-section (c-section) (1, 2). We report a case involving a woman who developed CCC of the abdominal wall 16 years after c-section, and describe the histological, immunohistochemical, and molecular features. Although the diagnostic criteria are well-defined, this diagnosis can be challenging and requires extensive sampling of the lesion, especially when cystic and benign-appearing glands are predominant.

## Case Study

**Figure 1:**
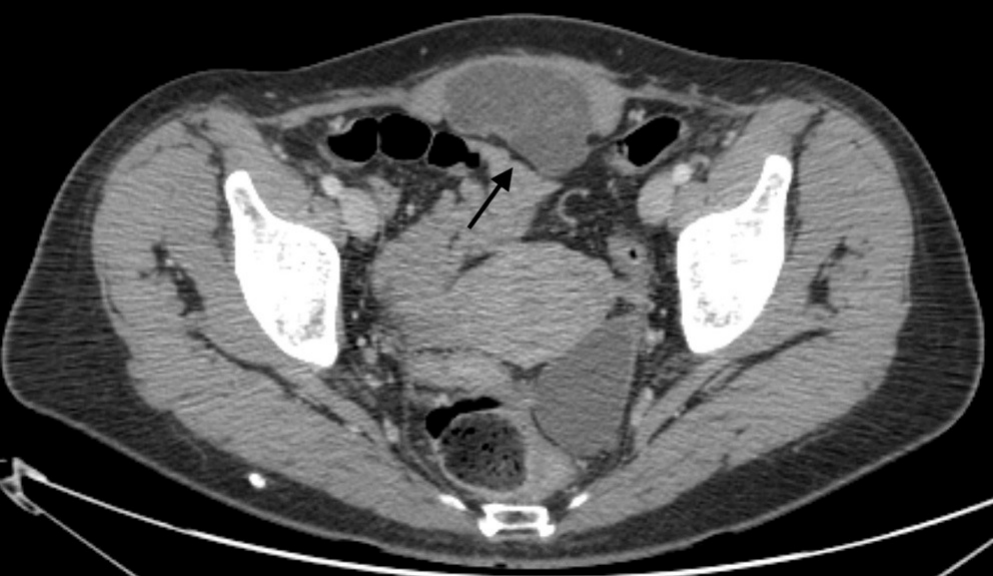
A solid, poorly vascularized mass, measuring 83 ×63 ×36 mm, with thin septa, is apparent in the rectus muscle of the abdomen in the suprapubic area (arrow).

In October 2018, a 52-year-old woman was admitted to the authors' institute for a subcutaneous mass that developed on her c-section scar. The patient had no history of endometriosis, cancer, or other relevant medical condition. Blood tests at admission were normal and tumor markers (CEA, CACA19.9) were negative. The patient underwent two cesarean deliveries via Pfannenstiel incision in 1999 and 2001, respectively. Physical examination revealed a subcutaneous smooth mass on the site of the c-section median scar. She had a non-painful, treatable abdomen. Ultrasound evaluation of the inferior abdomen revealed a solid mass, measuring 66 ×61 ×35 mm, adherent to the left lower rectus muscle, poorly vascularized, with microcysts in context. Computed tomography (CT) revealed evidence of a solid, poorly vascularized, partially cystic neoformation, with thin septa, localized in the left lower rectus muscle, with no other noteworthy findings (Figure 1). Unfortunately, a tru-cut biopsy was non-diagnostic; therefore, exploratory laparotomy was performed in November 2018. After peritoneal incision and lysis of peritoneal adhesions, the mass appeared to be localized to the rectal muscle of the abdomen, without involvement of the bladder and intestine. The uterus and ovaries did not exhibit any notable macroscopic pathological features. Intra-operatory pathological examination was performed, with a diagnosis of neoplasia of epithelial origin. The tumor was then removed *en-bloc* with parietal peritoneum and skin, with disease-free resection surgical margins. The abdominal wall was repaired through suture of the anterior and posterior aponeurosis and a polytetrafluoroethylene prothesis was placed. Subcutaneous drainage was removed on post-operative day 5 and the patient was discharged on day 6 after surgery with diagnosis of clear cell adenocarcinoma arising on a c-section scar. TC and PET 18-FDG negativity supported the diagnosis of primary carcinoma rather than metastasis. After 30 months of follow-up, the patient was in good condition and TC and PET 18-FDG were negative for disease relapse or metastasis, with negative serological tumor markers. The patient reported only some minor postoperative complications of abdominal wall repair. The patient perspective was taken in account when hysterectomy and bilateral salpingo-oophorectomy was considered.

**Figure 2:**
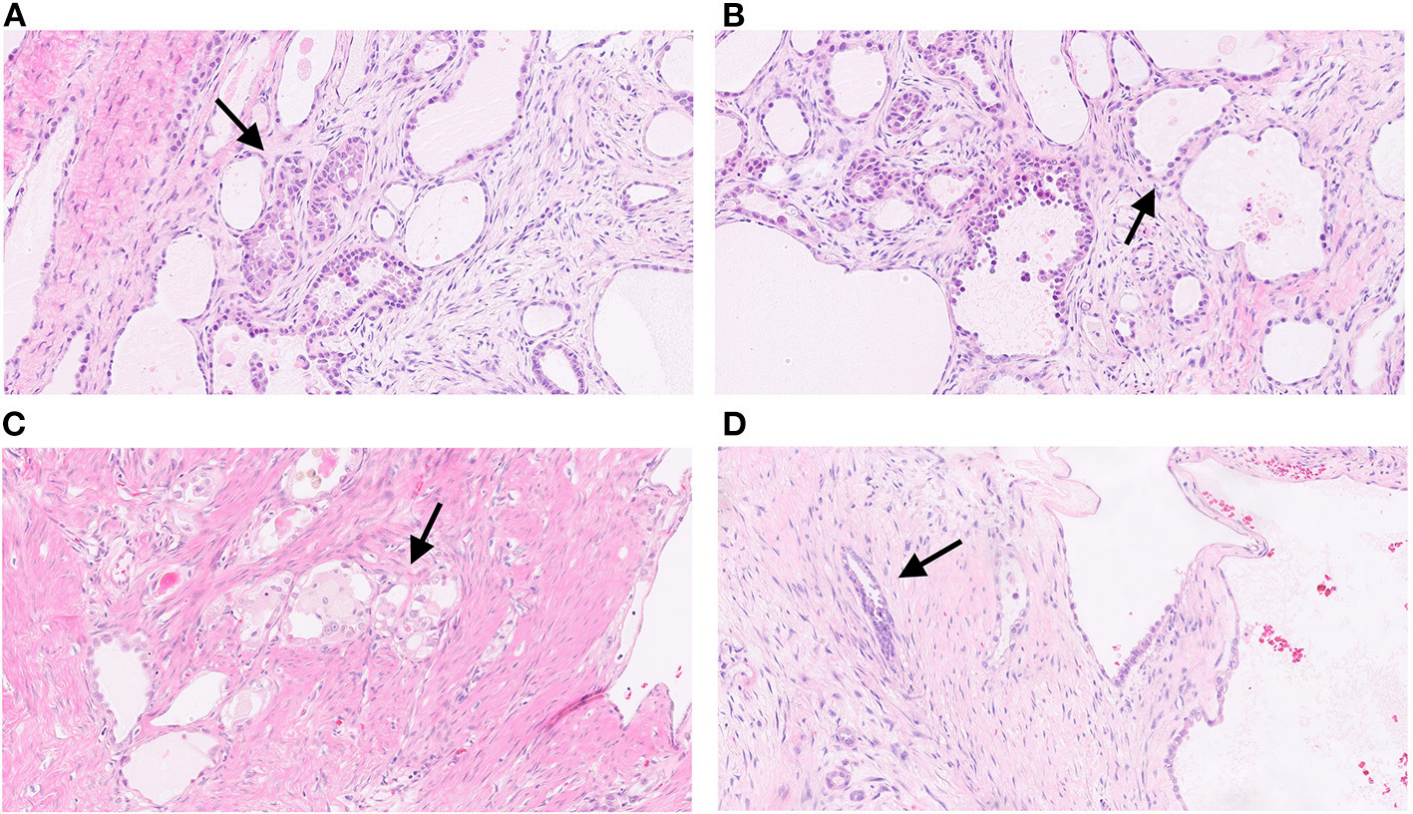
Histological images of the tumor. Carcinoma with papillary and tubule-cystic architecture. The cells exhibit clear and eosinophilic cytoplasm and hyperchromatic nuclei with mild to moderate atypia (arrow) (**A**; H&E, 5x). Dilated cysts lined by flattened cells with benign-appearing nuclei intermixed with atypical papillae (arrow) (**B**; H&E, 10x). Occasional small groups of clear cells (arrow) (**C**; H&E, 10x). Irregular infiltrating glands with dense fibrous stroma (arrow) (**D**; H&E, 10x).

**Figure 3:**
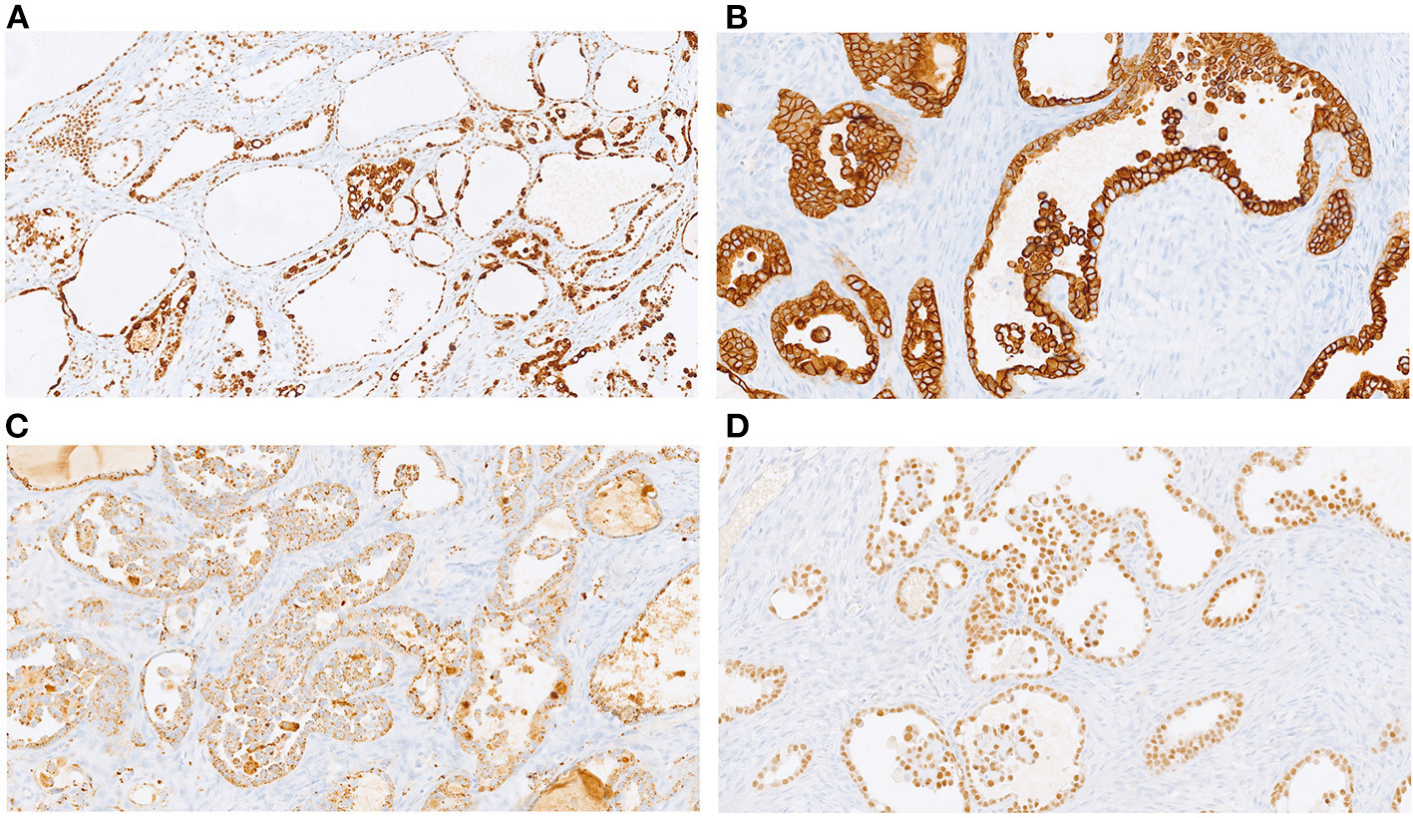
Immunohistochemical profile: diffuse expression of ARID1A (**A**, 20x), CK7 (**B**; 20x); napsin-A (**C**, 20x), and PAX8 (**D**; 20x).

Gross examination of the surgical sample revealed a well-defined nodular mass with cystic features. Histological analysis revealed carcinoma with papillary and tubule-cystic architecture. The cells exhibited clear and eosinophilic cytoplasm and hyperchromatic nuclei with mild to moderate atypia (Figure 2A). Moreover, intermixed with atypical papillae, dilated cysts lined by flattened cells with benign-appearing nuclei were observed (Figure 2B). Mitotic figures were scant. Occasional small groups of clear cells were observed (Figure 2C). No foci of benign endometrial glands were found; however, dense fibrous tissue and hemorrhagic areas were present mainly in the peripheral area of the lesion (Figure 2D). Immunohistochemical analysis revealed expression of CK7, PAX8, napsin-A, ARID1A, and membranous beta-catenin (Figure 3) in neoplastic cells, and negativity for ER, PR, CA125, CDX2, TTF1 and calretinin. Immunohistochemistry for p53 showed an heterogeneous expression in 20% of tumor cells so was considered TP53 wild type. Microsatellites were stable, with nuclear expression of MLH1, MSH2, MSH6, and PMS2. Real-time polymerase chain reaction (PCR) revealed the presence of the E545 mutation in *PIK3CA* in the helical domain of exon 9.

## Discussion

CCC is usually localized to the ovaries and endometrium. In the ovary, CCC represents 3–10% of epithelial carcinomas, depending on geographical location and ethnicity. Compared to serous carcinoma of the ovary, it usually appears at a younger age (55 vs. 64 years of age) and at a lower stage, which is usually associated with a better prognosis (1). In the abdominal wall, it is very rare, with approximately 30 cases described to date (2). As commonly reported for ovarian CCC, endometriosis is considered to be precursor lesion of CCC in the abdominal wall (3). The surgical scar from c-section is the most common site for extrapelvic endometriosis, with an incidence between 0.07 and 0.4%. In contrast, malignant transformation is a very rare event, accounting for <1% of cases (4), and CCC is the most frequent neoplastic lesion associated with endometriosis in the abdominal wall (5). Diagnosis of this malignancy requires the demonstration of both benign and neoplastic endometrial tissues in the tumor (6). In our case, endometriosis consisted primarily of stromal cells, without evidence of normal endometrial glands in the extensive sampling examined. Recently, beyond histological features, the molecular mechanisms underlying malignant transformation have been investigated. Anglesio et al. (7) described the presence of somatic mutations in deep infiltrating endometriosis without cancer, including mutations in *PIK3CA, KRAS*, and ARID1A. Loss of BAF250a (*ARID1A* gene) is also frequent in atypical endometriosis, suggesting its early contribution to carcinogenesis (8), and mutations in *PIK3CA* and *ARID1A* are common in CCC of the ovary (9), with *PIK3CA* being mutated in up to 35% of CCCs and *ARID1A* in 40%, which is associated with poor prognosis in stage I and II ovarian carcinomas. We detected E545 *PIK3A* mutation in exon 9 using real-time polymerase chain reaction, whereas the tumor exhibited preserved expression of the ARID1A protein according to immunohistochemical analysis. Loss of expression is correlated with inactivating mutations of *ARID1A* and poor prognosis in stage I/II ovarian CCC (10). Intact nuclear expression in our case appears to be consistent with the clinical history of the patient, who did not experience any recurrence or metastasis. As expected, β-catenin exhibited membranous expression on immunohistochemistry. Nuclear and aberrant expression, which is typical of ovarian endometrioid carcinoma, is highly correlated with *CTNNB1* mutation (11), but it has never been described in CCC (12). Despite the accumulation of molecular information, we did not find a consensus regarding specific treatment strategies. Radical surgery of a mass with disease-free margins is considered to be the best option. In some described cases, a bilateral annessectomy was performed (13); however, in our patient, surgical exploration, PET, and CT did not reveal any suspicious lesions at other sites. A multicenter study reported that adjuvant chemotherapy was not effective in stage I ovarian CCC (14). Several studies have considered experimental target therapies. PI3K pathway inhibitors may be considered for tumors with *PIK3CA* mutations (15). In our case, chemotherapy was not recommended by our oncologists or in other referral centers where the patient visited for a second opinion. Twenty-nine months after surgery, the patient is in a good health with no evidence of recurrence.

The increasing rate of c-sections requires more awareness of the existence of endometriosis in the scar site and the possibility—although highly limited—of malignant transformation. All reported cases suggest that a previous surgical procedure involving opening of the endometrial cavity may be a predisposing factor for the development of endometriosis implants in the abdominal wall. C-section is the most frequent surgical procedure associated with abdominal wall endometriosis, and also appears to be the most frequent surgery reported in cases of cancer derived from abdominal endometriosis implants.

## Data Availability Statement

The original contributions presented in the study are included in the article/supplementary material, further inquiries can be directed to the corresponding author/s.

## Ethics Statement

Written informed consent was obtained from the individual for publication of any potentially identifiable images or data included in this article.

## Author Contributions

MP, LC, ED, CD'A, CF, EA, and GG drafted the manuscript. CC and LM edited the manuscript. All authors were involved in the clinical care of the patient and approved the final version of the manuscript at the time of submission.

## Conflict of Interest

The authors declare that the research was conducted in the absence of any commercial or financial relationships that could be construed as a potential conflict of interest.

## Publisher's Note

All claims expressed in this article are solely those of the authors and do not necessarily represent those of their affiliated organizations, or those of the publisher, the editors and the reviewers. Any product that may be evaluated in this article, or claim that may be made by its manufacturer, is not guaranteed or endorsed by the publisher.
